# Alterations in the mucosal immune system by a chronic exhausting exercise in Wistar rats

**DOI:** 10.1038/s41598-020-74837-9

**Published:** 2020-10-21

**Authors:** Patricia Ruiz-Iglesias, Sheila Estruel-Amades, Mariona Camps-Bossacoma, Malén Massot-Cladera, Margarida Castell, Francisco J. Pérez-Cano

**Affiliations:** 1 Secció de Fisiologia, Departament de Bioquímica i Fisiologia, Facultat de Farmàcia i Ciències de l’Alimentació, Universitat de Barcelona (UB), Av. Joan XXIII 27-3, 08028 Barcelona, Spain; 2 Institut de Recerca en Nutrició i Seguretat Alimentària (INSA-UB), UB, 08921 Santa Coloma de Gramenet, Spain; 3 Centro de Investigación Biomédica en Red de Fisiopatología de la Obesidad y la Nutrición (CIBEROBN), Instituto de Salud Carlos III, Madrid, Spain

**Keywords:** Immunology, Physiology

## Abstract

Exhausting exercise can disturb immune and gastrointestinal functions. Nevertheless, the impact of it on mucosal-associated lymphoid tissue has not been studied in depth. Here, we aim to establish the effects of an intensive training and exhausting exercise on the mucosal immunity of rats and to approach the mechanisms involved. Rats were submitted to a high-intensity training consisting of running in a treadmill 5 days per week for 5 weeks, involving 2 weekly exhaustion tests. At the end, samples were obtained before (T), immediately after (TE) and 24 h after (TE24) an additional final exhaustion test. The training programme reduced the salivary production of immunoglobulin A, impaired the tight junction proteins’ gene expression and modified the mesenteric lymph node lymphocyte composition and function, increasing the ratio between Tαβ+ and B lymphocytes, reducing their proliferation capacity and enhancing their interferon-γ secretion. As a consequence of the final exhaustion test, the caecal IgA content increased, while it impaired the gut zonula occludens expression and enhanced the interleukin-2 and interferon-γ secretion. Our results indicate that intensive training for 5 weeks followed or not by an additional exhaustion disrupts the mucosal-associated lymphoid tissue and the intestinal epithelial barrier integrity in rats.

## Introduction

The mucosal immune system is the largest immune component of the body, shaped by the mucosa-associated lymphoid tissue (MALT) where about half of the whole lymphocyte population is found^1^. MALT cells are scattered along the surfaces of all mucosal tissues and constitute the starting point for a great number of immune responses because of its constant exposure to antigens. In addition, immune responses developed in a particular MALT structure will influence the immunity of the entire MALT due to its property of recirculating immune cells between mucosa and glands^2^. MALT comprises, among others, the salivary duct-associated lymphoid tissue (DALT) and the gut-associated lymphoid tissue (GALT), which defends the gastrointestinal tract against infections^1^. One of the main effector functions of the MALT is to produce and secrete immunoglobulin A (IgA)^1^.

It is well known that regular bouts of moderate-intensity exercise offers several long-term health benefits^3^, such as preventing, delaying or improving the prognosis of several chronic diseases^4^, and even cancer^5^, enhancing immunity^6^ and inducing benefits for the gastrointestinal (GI) tract^7^ and the gut microbiota^8^. However, chronic intensive exercise can induce adverse effects on health, such as oxidative stress, muscle damage and inflammation^9^, as well as immune^10^ and GI^11^ alterations. The increasing participation of the general population in endurance events over the last decades has raised concerns regarding the impact of prolonged overly intense exercise on immune and GI health^11,12^. Focusing on the immune system, the impact of physical activity also depends on the intensity and duration of the effort. Regular bouts of moderate exercise enhance immune function, whereas strenuous exercise may impair it, decreasing host protection accordingly and leading to a higher risk of GI and upper-respiratory tract infections (URTIs) 1–2 weeks after a competition^13^ and to a lower performance^14^. The appearance of GI symptoms related to excessive exercise has been reported to be 30–93% among distance runners and triathletes^15–17^. Most of them are mild and do not cause long-term health effects (epigastric pain, heartburn, nausea, vomiting, abdominal pain and diarrhoea), but oesophagitis, haemorrhagic gastritis, gastric ulcer, gastrointestinal bleeding and ischaemic bowel may involve severe medical complications^16,18,19^. The underlying mechanisms are not fully understood but this symptomatology seems to be mainly related to GI ischaemia, altered motility, malabsorption and neuroendocrine factors^11,20^.

The measurement of salivary IgA concentration in humans is one of the most used biomarkers to assess the effect of exercise on mucosal humoral immunity^21^. In particular, regular sessions of moderate exercise enhances salivary IgA secretion^22^, whereas prolonged periods of intensive exercise may decrease it, contributing, at least in part, to the higher susceptibility to infections observed in athletes^21^. On the other hand, exercise also impacts cellular immunity^21^. Most of the studies have assessed changes in blood lymphocytes, whereas only a few have focused on lymphoid compartments such as bone marrow, Peyer’s patches^21^, spleen, thymus^23^ and lymph nodes^24^. The changes in the proportion of lymphocyte populations that have been reported in such tissues may reflect a redistribution of cells among lymphoid tissues which must be mainly due to the release of stress hormones such as catecholamines and glucocorticoids^25^. In addition, changes in the functionality of natural killer (NK), T and B lymphocytes have been described^23,26^, as well as in the T helper (Th)1/Th2 cell balance^27^ in both blood and lymphoid tissues. Previous studies have evidenced the effect of exercise on axillary, inguinal and submandibular lymph nodes’ specific immunity^24^, but, to our knowledge, the impact of high-intensity exercise in the GALT, and particularly in mesenteric lymph nodes (MLNs), remains uncertain. MLNs belong to the organized GALT and play an important role in the development of local immune responses in the gut^28^. Therefore, alterations in MLN lymphocyte (MLNL) composition and function may contribute to the explanation of the mechanisms underlying exercise-induced gastrointestinal syndrome.

Despite the existing research, the isolated role of exercise in disrupting GI and immune function has been challenged lately, since increasing evidence suggests that many other uncontrolled factors, such as anxiety, sleep deprivation, travel, nutritional deficits, environmental extremes and exposure to pathogens by attending a mass participation event may be involved^6^. Therefore, a well-controlled animal model of intensive exercise may be useful to elucidate the impact of exercise per se on the immune system, and, more specifically, on the MALT. Previously, we have characterized the alterations occurring in the innate and adaptative immune system as well as in the oxidative status in high-intensity trained rats^23,26,29^. The present study aimed to establish the impact of intensive training and exhaustion exercise on the mucosal immune system functionality of rats and to approach the mechanisms involved.

## Results

### Performance

The high-intensity training programme involved 5 days of training per week for 5 weeks: 3 regular trainings on Tuesday, Wednesday and Thursday, and an exhaustion test (ET) every Monday (M) and Friday (F), in which maximum running time indicated rat physical performance (Table 1). Fridays’ performance was better than that on Mondays (p < 0.05 for weeks 3 and 4). Rats supported a maximum time of about 25 min on the second Friday, achieving a maximum speed of about 59.0–71.8 m × min^-1^. Later, in weeks 3–5, the performance achieved on Monday was lower than that on Mondays from previous weeks (p < 0.05), which was also observed on the last Friday in comparison with the previous ones (p < 0.05). An additional ET was performed after the 5-week high-intensity training programme, where trained rats ran for 32.07 ± 1.50 min (mean ± standard error).

**Table 1:** Maximum running time lasted in the Monday and Friday exhaustion tests performed throughout the high-intensity exercise training programme.

Week	Maximum running time (min)		
	Monday ET	Friday ET	p value
1	23.83 ± 0.51	24.70 ± 0.55	NS
2	23.83 ± 0.61	25.17 ± 0.81	NS
3	22.26 ± 0.70*	24.22 ± 0.92	0.042
4	21.18 ± 0.94*	23.48 ± 0.59	0.003
5	21.48 ± 0.76*	22.30 ± 0.67#	NS

*ET* exhaustion test, *NS* no statistically significant differences detected. Data are expressed as mean ± SEM (n = 8). Statistical differences (paired Student *t* test): significant differences between consecutive Monday ET and Friday ET are included in the table (p < 0.05); *significant differences vs the first two Monday ETs (p < 0.05); ^#^significant differences vs the first three Friday ETs (p < 0.05).

### Body weight and food efficiency

**Figure 1:**
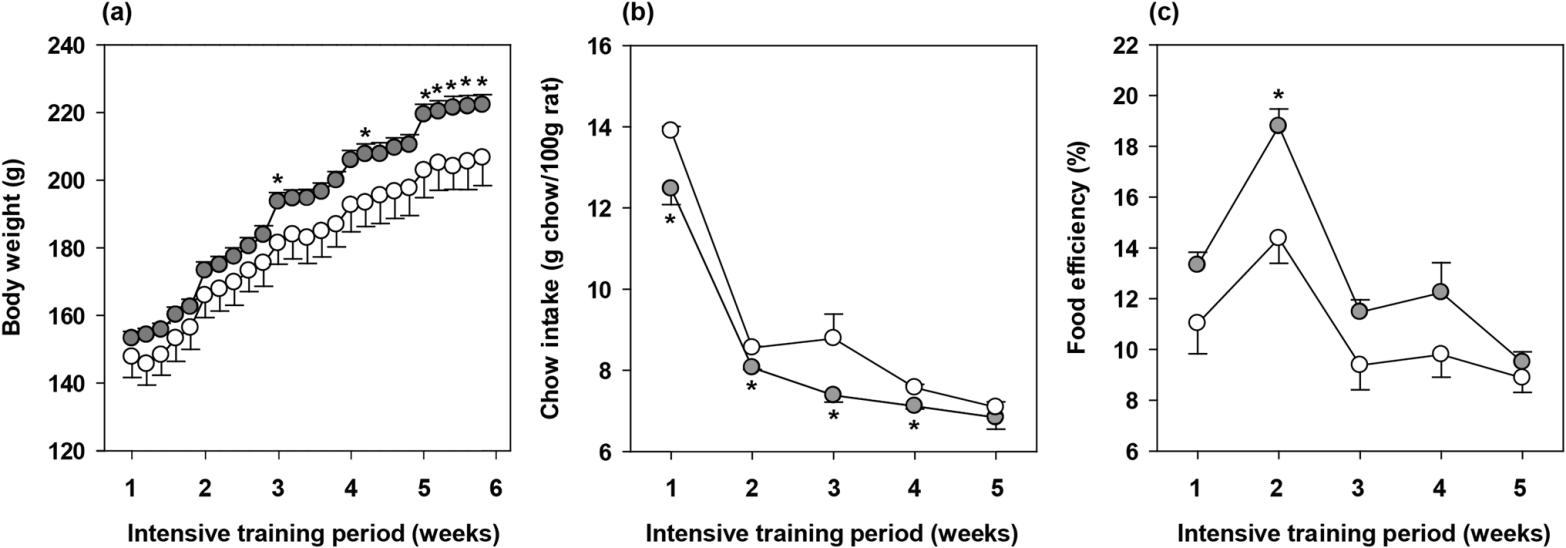
Body weight (**a**), chow intake (**b**), and food efficiency (**c**) throughout the intensive training period. The sedentary (SED) group is represented by white symbols (○) and the runner group by grey symbols ( ). Data are expressed as mean ± standard error (n = 8 animals for the SED group and n = 24 animals for the runner group in (**a**); n = 3 cages for the SED group and n = 8 cages for the runner group in **b**,**c**). Statistical differences (Student’s t test): *p < 0.05 vs SED group.

Body weight and chow intake were monitored throughout the 5-week training programme (Fig. 1a,b) in both runners and sedentary (SED) rats. Although the body weight was similar during the first weeks of training, runner animals showed a higher body weight than SED animals on the last days of the study (p < 0.05). This increase was not associated with a higher chow intake, quite the opposite: runner animals showed a lower weekly chow intake than SED rats from the beginning of the training programme (p < 0.05 in weeks 1–4).

Body weight gain and chow intake allowed the calculation of the week-long food efficiency (Fig. 1c). The pattern of food efficiency progression during the study was similar between SED and runner animals, substantially increasing during the first 2 weeks, probably due to the young age of the animals. The high-intensity training tended to increase the food efficiency throughout the study, the difference being statistically significant only in the second week of the training programme (p = 0.008).

### Salivary gland immunoglobulins

After the 5-week intensive training, in order to assess the mucosal immune status at different time points, runner rats were divided into 3 groups according the conditions of sample collection: T group, whose sample were collected one day after performing a regular training, TE group, whose samples were obtained immediately after an additional final exhaustion test, and TE24 group whose samples were collected 24 h after the additional final exhaustion test. The results from these rats were compared with those obtained from matched sedentary rats (SED).

**Figure 2:**
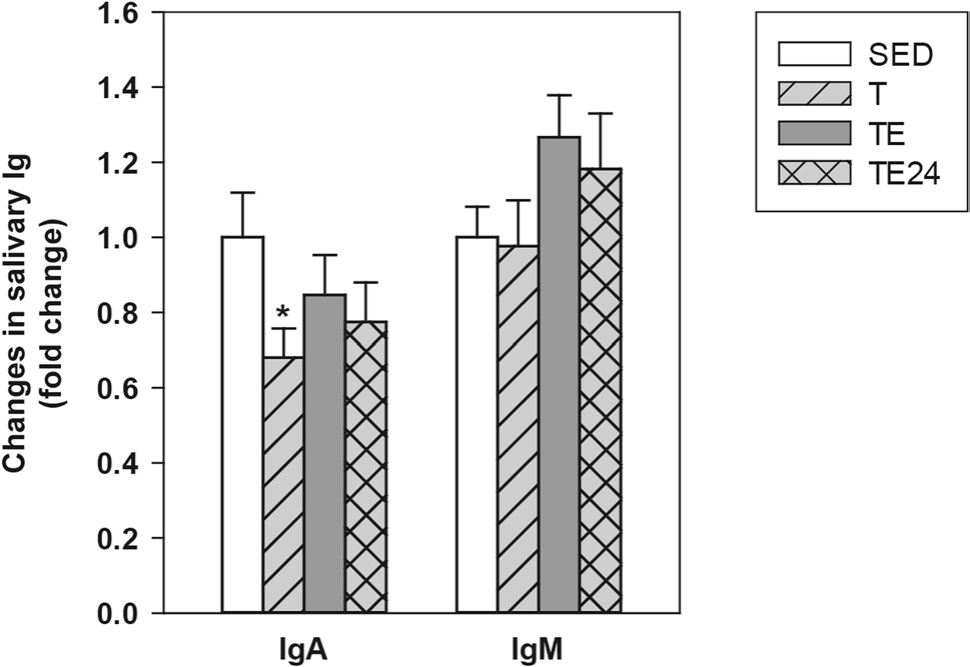
Changes in submaxillary salivary gland IgA and IgM concentration compared to the sedentary group. *SED* sedentary rats, *T* trained rats, *TE* T rats immediately after a final exhaustion test, *TE24* TE rats 24 h after the final exhaustion test. Data are expressed as mean ± standard error (n = 8). Statistical differences (one-way ANOVA followed by post-hoc Tukey test): *p < 0.05 vs SED group.

Content of IgA and IgM was determined in submaxillary salivary glands (SMGs) (Fig. 2). The intensive training induced a decrease in the IgA content in this compartment (p = 0.05, T group vs SED group) that was not statistically significant after carrying out the additional final ET. Neither training nor exhaustion induced significant changes in salivary IgM concentration, although exhaustion tended to increase it in comparison to sedentary animals (p = 0.098, TE group vs SED group).

### Intestinal immunoglobulins

**Figure 3:**
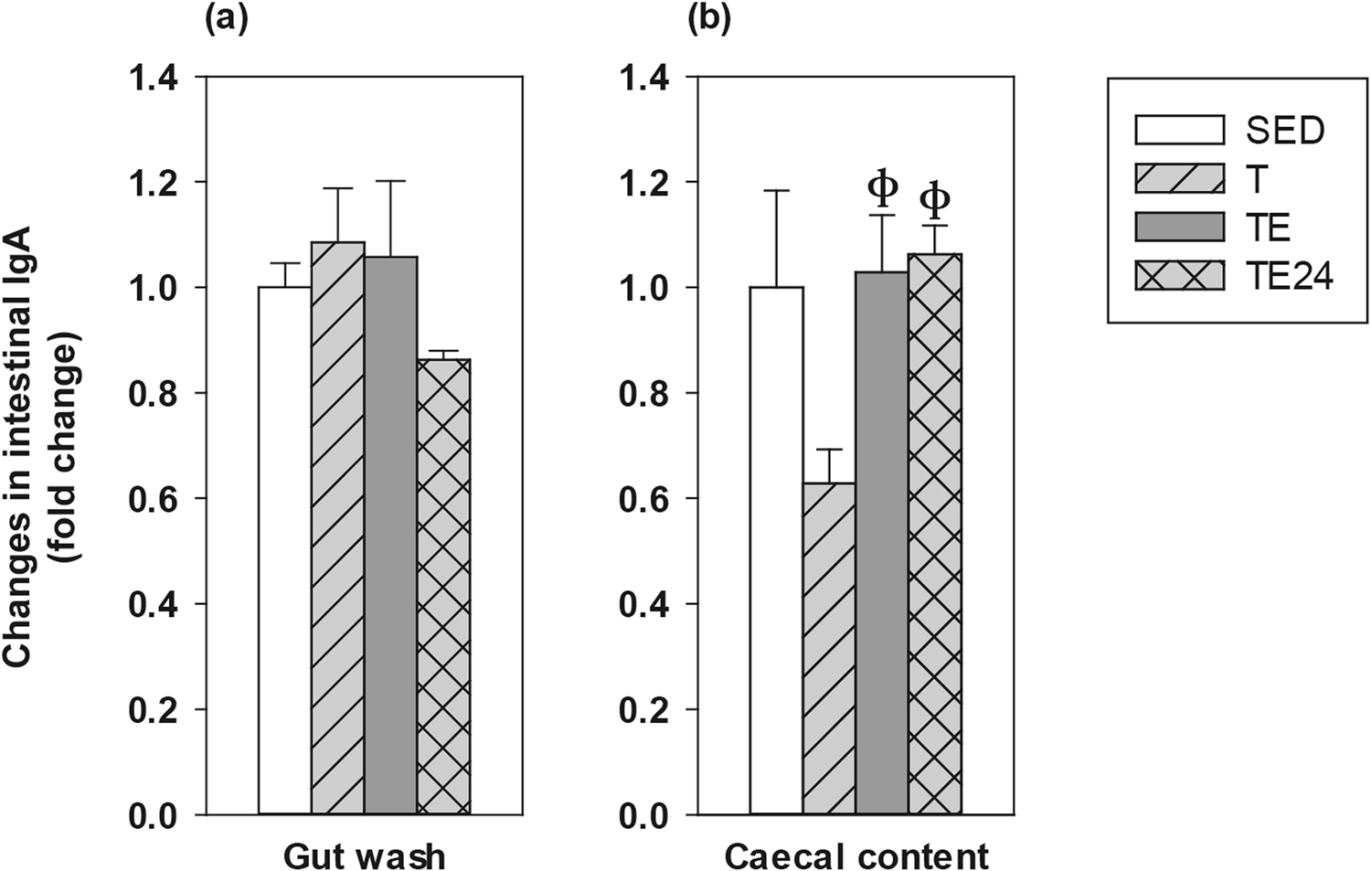
Changes in gut wash IgA (**a**), and caecal content IgA (**b**) concentration compared to the sedentary group. *SED* sedentary rats, *T* trained rats, *TE* T rats immediately after a final exhaustion test, *TE24* TE rats 24 h after the final exhaustion test. Data are expressed as mean ± standard error (n = 8). Statistical differences (Kruskal–Wallis followed by Mann–Whitney *U* test): ^ф^p < 0.05 vs*.* T group.

Intestinal IgA concentration was determined in gut washes (GWs) from the small intestine and in caecal content (CC) (Fig. 3a,b). In the GWs, neither training nor exhaustion induced changes in IgA content, although values tended to decrease 24 h after the final exhaustion test without reaching significance. With regard to the CC, IgA levels tended to decrease after 5 weeks of intensive training (T group) but, after the final exhaustion test, there was a higher IgA content that remained for at least 24 h (p = 0.018, T group vs TE group; p = 0.009, T group vs TE24 group).

### Small intestine gene expression and gut permeability

Alterations due to the intensive training programme and the final exhaustion in the gene expression of proteins involved in the gut homeostasis, including tight junction proteins (such as occludin, claudin-2, -4, zonula occludens-1, -2) and proteins involved in the B cell differentiation, IgA transcytosis and lymphocyte gut homing, were established (Table 2).

**Table 2:** Changes in gene expression of some molecules in small intestine compared to the sedentary group.

Gene	SED	T	TE	TE24
*Oclud*	1 ± 0.09	0.81 ± 0.04*	0.86 ± 0.08	1.09 ± 0.01^ф^
*Cldn-2*	1 ± 0.17	2.97 ± 0.43*	3.98 ± 0.70*	2.70 ± 0.54*
*Cldn-4*	1 ± 0.13	0.62 ± 0.05*	0.97 ± 0.07^ф^	1.19 ± 0.23^ф^
*ZO-1*	1 ± 0.11	1.12 ± 0.10	1.76 ± 0.23*^ф^	1.25 ± 0.16^λ^
*ZO-2*	1 ± 0.05	0.88 ± 0.03	0.75 ± 0.07*^ф^	1.10 ± 0.05^фλ^
*pIgR*	1 ± 0.15	1.08 ± 0.08	0.83 ± 0.13	0.87 ± 0.14
*TGF-β*	1 ± 0.20	0.64 ± 0.09	0.75 ± 0.13	0.60 ± 0.04
*RAR-α*	1 ± 0.07	0.83 ± 0.05	0.75 ± 0.04*	0.89 ± 0.06
*CCL25*	1 ± 0.08	1.02 ± 0.06	0.97 ± 0.11	0.96 ± 0.10

*SED* sedentary rats, *T* trained rats, *TE* T rats with a final exhaustion test, *TE24* TE rats 24 h after the final exhaustion test. Data are expressed as mean ± standard error (n = 8). Statistical differences (Kruskal–Wallis followed by Mann–Whitney *U* test): *p < 0.05 vs SED group; ^ф^p < 0.05 vs T group; ^λ^p < 0.05 vs TE group.

In the case of the tight junction proteins, the 5-week training programme decreased occludin (Oclud) and claudin (Cldn)-4 expression (p = 0.044 and p = 0.007, respectively, T group vs SED group), although both levels increased after performing the additional final ET. The claudin-4 expression found in the TE24 group inversely correlated with the performance achieved in the additional final ET (R = − 0.928, p = 0.008). In contrast, claudin-2 expression increased due to both intensive training and final exhaustion (p < 0.001 and p < 0.001, T group and TE group vs SED group), and these higher levels were maintained for at least 24 h (p = 0.011, TE24 group vs SED group).

In samples obtained immediately after the final exhaustion test (TE group) the expression of zonula occludens (ZO)-1 increased (p = 0.023, TE group *vs.* SED group) and that of ZO-2 decreased (p = 0.004, TE group *vs.* SED group) with respect to the sedentary rats. Both changes were normalized 24 h later.

With regard to the gene expression of proteins involved in B cell differentiation, IgA transcytosis and gut homing, no changes were found for polymeric immunoglobulin receptor (pIgR), transforming growth factor (TGF)-β1 and chemokine (C–C motif) ligand 25 (CCL25) due to the intensive training and/or exhaustion in comparison to sedentary animals. Nevertheless, the additional final ET decreased the retinoic acid receptor (RAR)-α gene expression. When considering the association of this results with the performance, there was an inverse correlation between the performance achieved in the additional final exhaustion test and the intestinal gene expression of TGF-β in the TE24 group (R = − 0.775; p = 0.041).

**Figure 4:**
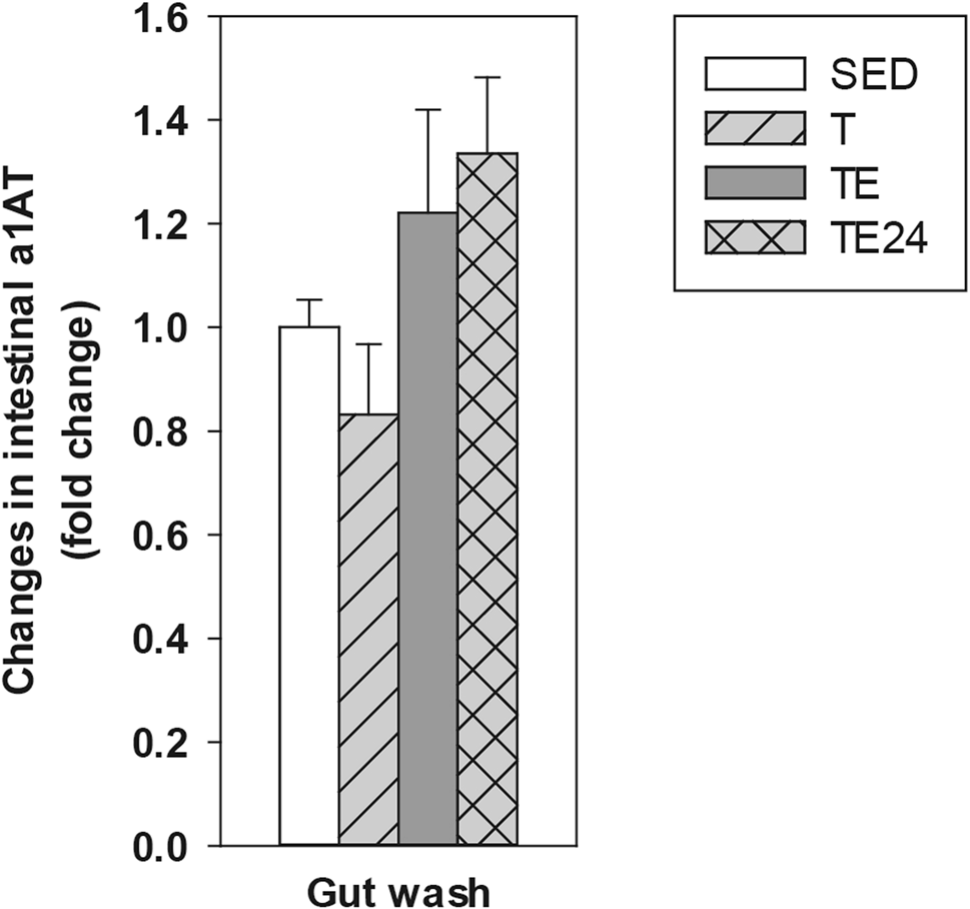
Changes in gut wash alpha-1-antytripsin (a1AT) concentration compared to the sedentary group. *SED* sedentary rats, *T* trained rats, *TE* T rats immediately after a final exhaustion test, *TE24* TE rats 24 h after the final exhaustion test. Data are expressed as mean ± standard error (n = 8).

On the other hand, alpha-1-antitrypsin (a1AT) concentration in GW was determined in order to assess changes in the gut paracellular permeability (Fig. 4). Although no significant changes in this marker were observed due to the 5 weeks of intensive training, the a1AT content in the GW tended to increase 24 h after carrying out the additional final ET (p = 0.092, TE24 group vs T group).

### MLN lymphocyte composition

**Figure 5:**
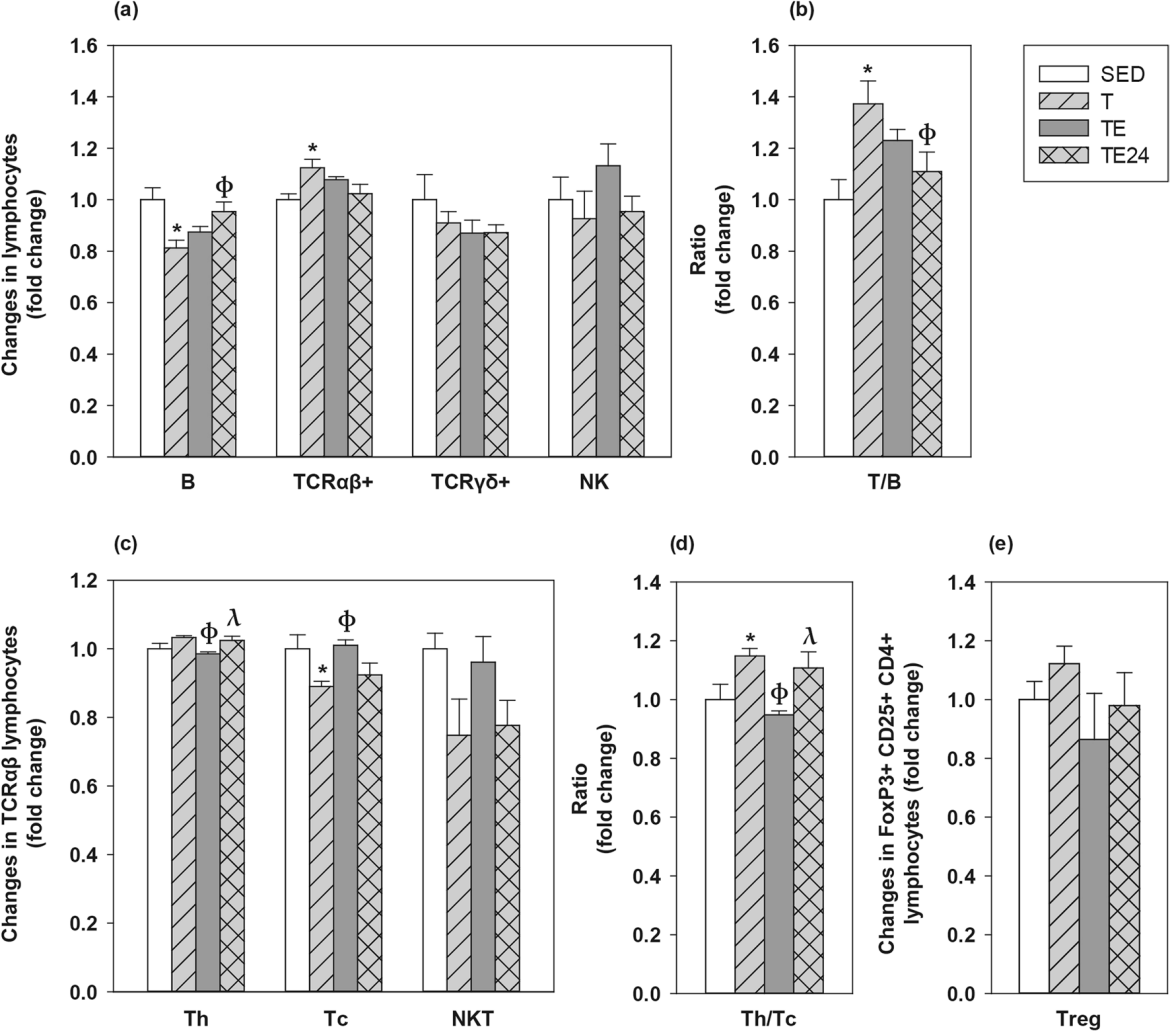
Changes in the proportion of mesenteric lymph node lymphocytes compared to the sedentary group. Main lymphocyte subsets (**a**); Tαβ/B lymphocytes ratio (**b**); main Tαβ subsets (**c**); Th/Tc ratio (**d**) and Treg proportion (**e**). *SED* sedentary rats, *T* trained rats, *TE* T rats with an additional final exhaustion test, *TE24* TE rats 24 h after the additional final exhaustion test. Data are expressed as mean ± SEM (n = 8). Statistical differences (one-way ANOVA followed by post-hoc Tukey test for (**a**,**b**) and Kruskal–Wallis followed by Mann–Whitney *U* test for **c**–**e**): *p < 0.05 vs SED group; ^ф^p < 0.05 vs T group; ^λ^p < 0.05 vs TE group.

The influence of intensive training and the final exhaustion on the MLNL composition was also assessed (Fig. 5). In SED animals, MLNL included 33.10 ± 1.53% of B cells (CD45RA+), 57.45 ± 1.31% of Tαβ cells (TCRαβ+), 1.99 ± 0.19 of Tγδ cells (TCRγδ+) and 0.44 ± 0.04% of NK cells (CD161b+). Among all Tαβ cells, 75.04 ± 1.17% were Th (CD4+CD161b− in TCRαβ+), 25.13 ± 1.03% Tc (CD8+CD161b− in TCRαβ+) and 1.09 ± 0.05% NKT (CD161b+ in TCRαβ+) cells. The proportion of regulatory T (Treg) cells within CD4+ lymphocytes (CD25+FoxP3+ in CD4+) was 1.79 ± 0.11%.

The 5-week intensive training programme decreased the proportion of MLN B cells (p = 0.008, T group vs SED group) whereas it increased that of Tαβ lymphocytes (p = 0.044, T group vs SED group) (Fig. 5a). Consequently, the T/B cell ratio increased in the T group with respect to the SED group (p = 0.011) up to about 40% (Fig. 5b). After the final exhaustion test, the B and Tαβ cell proportions did not significantly differ from those of SED animals. No exercise condition modified the Tγδ and NKT cell proportions; however, in the TE group there was an inverse correlation between the time supported in the additional final exhaustion test and the proportion of MLN Tγδ cells (R = -0.757; p = 0.049).

With regard to the main Tαβ subsets, the MLN Tc cell proportion decreased in the T group (p = 0.008) compared to SED animals, which increased the Th/Tc ratio up to 20% (Fig. 5c,d). However, the proportion of Tc cells increased immediately after carrying out the additional final ET (p = 0.004, TE group vs T group), when there was a significant reduction of Th cell proportion (p = 0.003, TE group vs T group) and, as a result, a lower Th/Tc ratio than that observed in just trained rats (p = 0.004, TE group vs T group). All these changes concerning Tc and Th proportions in TE group were restored 24 h later, in the TE24 group.

Although none of the exercise conditions studied significantly modified the percentage of MLN Treg cells (Fig. 5e), there was an inverse correlation between the Treg proportion and the performance in the final exhaustion test (R = − 0.986, p < 0.001 in the TE group and R = − 0.874, p = 0.005 in the TE24 group).

### MLN lymphocyte functionality

**Figure 6:**
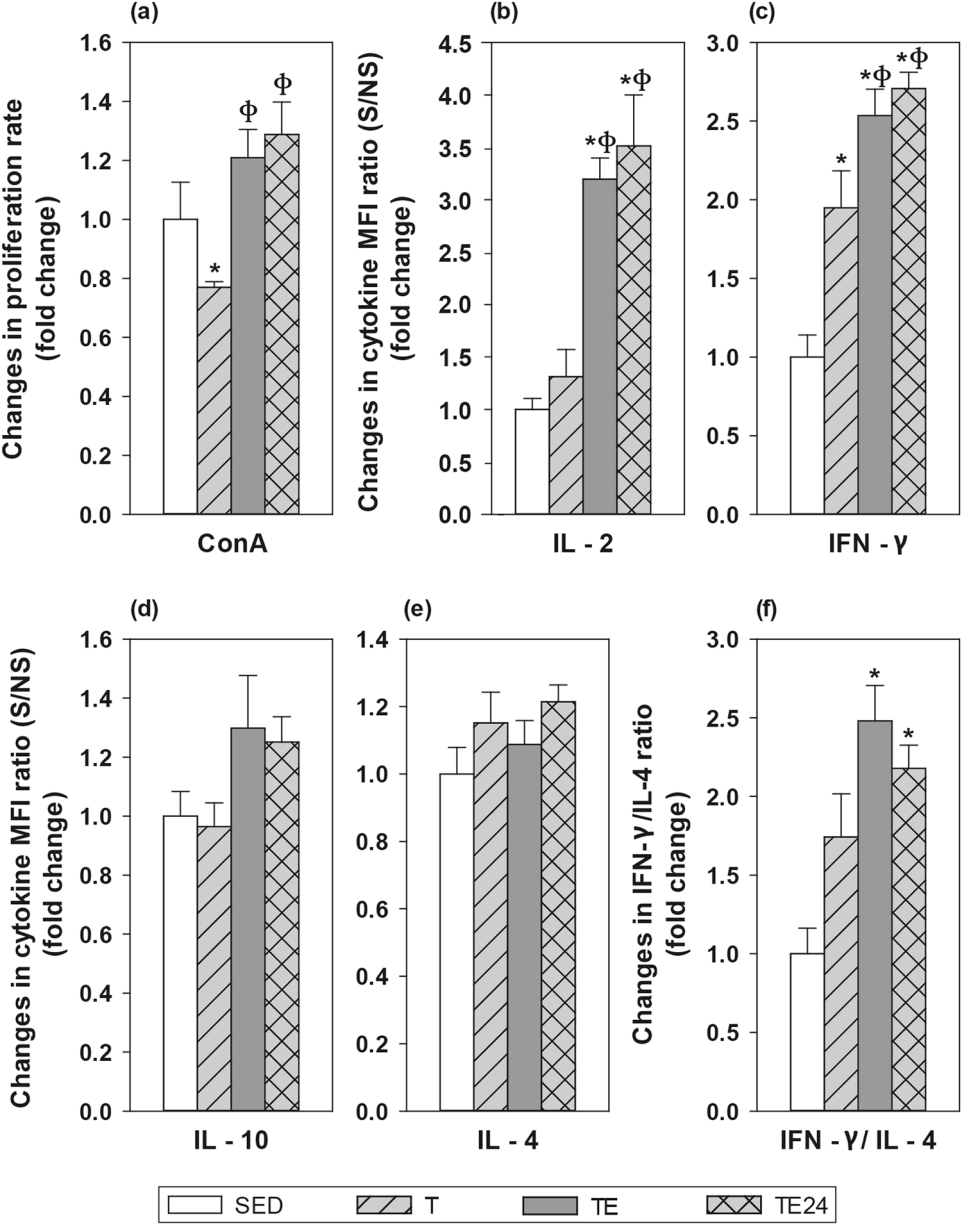
Changes in the proliferative response (**a**) and cytokine concentration released (**b**–**f**) by mesenteric lymph node lymphocyte stimulated by concanavalin A (ConA) compared to the sedentary group. *SED* sedentary rats, *T* trained rats, *TE* T rats with a final exhaustion test, *TE24* TE rats 24 h after the final exhaustion test. Data are expressed as mean ± standard error (n = 8). Statistical differences (one-way ANOVA followed by post-hoc Tukey test for **b**,**d**, and Kruskal–Wallis followed by Mann–Whitney *U* test for **a**,**c**,**e**): *p < 0.05 vs SED group; ^ф^p < 0.05 vs T group.

The proliferative response of MLN T-cells was established after concanavalin A (ConA) stimulation (Fig. 6a). After 5 weeks of intensive training, a lower proliferation capacity than that in the SED group was observed (p = 0.045, T group vs SED group). Nevertheless, it was overcome after carrying out the additional final ET (p = 0.006, TE group vs T group; p = 0.003, TE24 group vs T group).

Cytokine secretion was also quantified in MLNL culture supernatants (Fig. 6b–f). In SED animals, MLNL secreted 356.18 ± 50.94 pg/mL of interleukin (IL)-2, 1624.30 ± 469.29 pg/mL of interferon (IFN)-γ, 73.67 ± 12.92 pg/mL of IL-10, and 1.33 ± 0.19 pg/mL of IL-4 (mean ± standard error). The intensive training programme doubled the secretion of IFN-γ in MLNL (p = 0.005, T group vs SED group), and the final exhaustion test enhanced it even further (p < 0.001, TE group vs SED group), remaining elevated for at least 24 h (p < 0.001, TE24 group vs SED group). The final exhaustion test also increased the secretion of IL-2 in the TE (p = 0.003 vs SED group, p = 0.006 vs T group) and TE24 groups (p = 0.001 vs SED group, p = 0.002 vs T group). There were no significant changes regarding the secretion of IL-4 and IL-10 by MLNL, nevertheless, the levels of IL-10 positively correlated with the performance in the TE group (R = 0.921, p = 0.026).

The IFN-γ/IL-4 ratio was calculated in order to assess the Th1/Th2 balance (Fig. 6f). Both the TE and TE24 groups showed a significantly higher IFN-γ/IL-4 ratio (p = 0.001 and p = 0.006, respectively, vs SED group).

**Figure 7:**
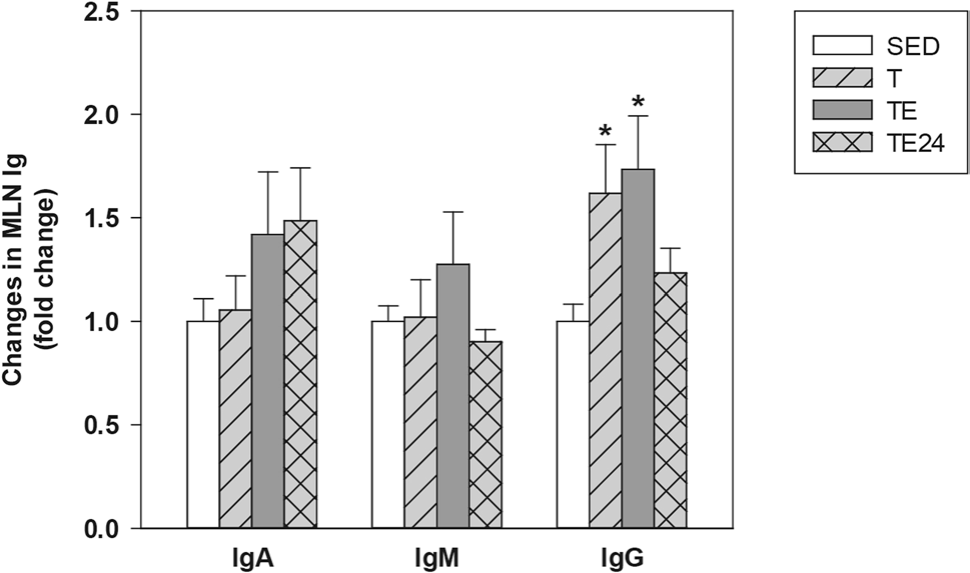
Changes in the IgA (**a**), IgM (**b**) and IgG (**c**) concentration in mesenteric lymph node lymphocyte supernatants compared to the sedentary group. *SED* sedentary rats, *T* trained rats, *TE* T rats with a final exhaustion test, *TE24* TE rats 24 h after the final exhaustion test. Data are expressed as mean ± standard error (n = 8). Statistical differences (Kruskal–Wallis followed by Mann–Whitney *U* test): *p < 0.05 vs SED group.

Finally, the in vitro ability for antibody production was determined in supernatants from non-stimulated MLNL (Fig. 7). In SED animals, MLNL in the assayed conditions produced 74.87 ± 10.39 ng/mL of IgA, 20.75 ± 5.54 ng/mL of IgM, and 17.81 ± 2.63 ng/mL of IgG (mean ± standard error). No changes were found in IgA and IgM secretion due to the training or the exhaustion test; however, IgG production remarkably increased in both the T and the TE groups (p = 0.014 and p = 0.009, respectively, vs SED group).

## Discussion

Nowadays, it is widely accepted that strenuous exercise impairs the immune system and promotes gastrointestinal symptoms^10,11^. A growing amount of research is investigating the underlying mechanisms of exercise-induced gastrointestinal syndrome^11,30,31^ and conclude that it is likely that this has a multifactorial cause, involving circulatory, enteric and immune alterations, as well as environmental triggers^11^. Efforts to link this symptomatology with intestinal barrier disruption due to splanchnic ischaemia have been relatively successful; nevertheless, the available literature on the mucosal immune system’s contribution to these symptoms remains quite controversial. In previous studies, we demonstrated that rats submitted to intensive training and exhausting exercise exhibited alterations in both the innate and the acquired immunity^23,26^. The current study aimed to establish the effects of 5 weeks of intensive training on the mucosal immune system in rats and to elucidate the mechanisms underlying these effects.

Rats intensively trained for 5 weeks showed a better performance in the first 2 weeks but, in the last 2 weeks there was a clear lower performance, thus suggesting that animals may have achieved overtraining syndrome, which appears when the training workload exceed the body’s ability to recover and this results, among other consequences, in a decrease in expected levels of performance^10^. Those runner animals showed a higher body weight gain, a lower chow intake and better food efficiency than sedentary animals throughout the training programme. This higher body weight (and consequently better food efficiency) could be due to an increase in muscle mass^32^ and/or bone mass^33^ and a decrease in fat content, as it has already been reported in female trained rats^34^ and mice^35^. However no increases in body weight have been reported in male trained rodents^34–37^. These differences associated with rat gender deserve new studies aiming to establish the relationship between food intake, body weight and fat and muscle tissues in both male and female intensively trained rats.

Tight junction (TJ) proteins play a key role in maintaining the intestinal barrier function by regulating paracellular transport and secretion/absorption mechanisms. Claudins and zonula occludens proteins are vital for the TJ assembly and resistance, while occludin has a less vital regulatory role in the TJ^30^. There is evidence that the intestinal ischaemia-induced by exercise leads to an impairment of the gut epithelial barrier integrity, by means of TJ protein phosphorylation, disrupting the gut paracellular permeability^30,31^. Here, we found a decrease in occludin and claudin-4 gene expression due to the intensive training programme that could result in overtraining. Whereas these results agree with previous studies that found reduced occludin levels in intestinal cells in an in vitro heat stress model aiming to simulate exercise stress and recovery^38^, they disagree with others that found similar expression levels in endurance exercise-trained rats and their sedentary counterparts^39^, or even an increased occludin expression in obese rats submitted to high-intensity interval training^40^. To the best of our knowledge, changes in claudin-4 expression due to exercise have not been previously reported and they could have a relevant impact on GALT’s functionality, since this TJ protein appears to participate in M cell transcytosis to intake intestinal antigens^41^. On the other hand, in line with previous research on endurance swimming training in rats^42^, both training and exhaustion induced an increase in claudin-2 expression, which, together with claudin-1 and claudin-3, is essential to form the TJ seal^30^. Nevertheless, claudin-2 is a pore-forming claudin^43^ and its upregulation has been associated with an undergoing intestinal inflammatory process that contributes to leak-flux diarrhoea^44^, which is a frequently reported symptom in endurance athletes^11,31^. Therefore, such increase in claudin-2 could be partially responsible for these gastrointestinal symptoms. Zonula occludens-1 was also upregulated after performing the final additional exhaustion test, in agreement with other authors^39,40,42^, while zonula occludens-2 was downregulated. Despite all these changes in TJ proteins’ gene expression, we did not observe a marked effect of exercise on intestinal permeability assessed by a1AT concentration in GW, but only a trend of increasing permeability after performing the additional final exhaustion test. Nevertheless, a previous study carried out in trained men found normal levels of faecal a1AT while zonulin’s were slightly above normal^45^. Whereas alpha-1-antitrypsin function as a marker of increased intestinal permeability is based on its high concentration in serum and its extravasation into the gut when the epithelial barrier function is impaired, zonulin plays a specific physiological role in disassembling intercellular TJ and altering the paracellular transport of fluids, ions, macromolecules and leukocytes^46^. For that reason, zonulin has recently become one of the most valid surrogate markers to estimate intestinal permeability^31^. In this regard, a recent study compares the intestinal permeability assessment with the traditional assay based on the differential sugar absorption method (lactulose/mannitol test) and zonulin concentration in stool; both were higher in professional athletes than in healthy non-athletes but no significant association between them was found^47^. Therefore, although a growing number of studies using both traditional permeability assays and biomarkers of intestinal inflammation quantification conclude an increased gut permeability after performing intensive exercise^11,48^, further studies may clarify the remaining controversies of these results and find the most accurate method to monitor this variable.

With regard to the effects of exercise on the MALT, the majority of exercise studies focused on salivary secretory IgA (SIgA) as a marker of MALT functionality^6,49^. It has been reported that intensive exercise and overtraining produces, in most studies, a decline in salivary SIgA levels^49,50^. The SIgA decrease reported in these studies has been attributed to a reduction in the pIgR expression, an essential receptor involved in the IgA transcytosis across the epithelial cells^52^, which could be due to either prolonged sympathetic nervous system overactivation and elevated cortisol concentration^50^ or indirectly mediated by cytokines induced by exercise^52^. In agreement with these previous studies^49,50^, we found a reduced IgA content in the whole submaxillary salivary gland tissue, that was not counteracted by higher IgM levels^2^, which has been reported to be both reduced^56^ and increased^57^ after performing intensive exercise. Although IgA content in salivary glands is not the same that IgA content in the saliva, it indicates the ability to synthetize this mucosal antibody which will be later submitted to transcytosis and exocytosis, throughout pIgR, to the ducts and eventually will be found in the saliva. Regarding another mucosal compartment, we found no changes either in the pIgR expression in the wall of small intestine and in the IgA secreted in the intestinal lumen measured in GW. Therefore, further studies must clarify how exercise particularly affects salivary glands and the IgA synthesis, exocytosis and transcytosis therein.

On the other hand, other alterations by the intensive training and the possible overtraining were found in molecules expressed in the GALT. Thus, there was a reduction in RAR-α gene expression due to exhaustion (as observed immediately after the exhaustion test) and an inverse correlation between TGF-β levels and the time supported in the additional final exhaustion test (found 24 h later), meaning that those rats running longer had lower TGF-β expression. Both RAR-α and TGF-β are involved in IgA+ B cell differentiation and gut homing, and their decreased expression after exhaustion could be translated in a lower concentration of intestinal IgA later, as we observed 24 h after exhaustion without reaching significance.

At caecal level, intensive training tended to decrease the caecal IgA content but the final exhaustion test raised it. This increase could be partially due to changes in the caecal microbiota induced by the exhaustion that might involve the release of IgA previously coated to bacteria. In fact, although benefits of moderate exercise in the gut microbiota have been reported^8,57^, there is still limited knowledge regarding exhausting exercise-induced microbiota alterations. In this context, whereas some authors reported that high-intensity interval training could overcome some of the detrimental effects in the gut microbiota caused by high-fat diet-induced obesity in mice^58^, others have described that healthy mice submitted to a 4-week intensive swimming training hosted a less diverse microbiota^59^. Thus, further studies should be carried out to clarify the effects of intensive exercise and exhaustion in the gut microbiota composition and functionality, as well as the changes induced by exhaustion in the proportion of bacteria coated to IgA.

To take a more in-depth look into understanding exercise-induced GALT alterations, changes in the composition and functionality of mesenteric lymph node lymphocytes were assessed. Neither training nor exhaustion modified the proportion of the minority lymphocyte populations NK, Tγδ and Treg cells. However, we found an inverse correlation between the time supported in the final exhaustion test and the percentage of MLN Tγδ cells in just exhausted rats, probably due to cell apoptosis, in agreement with previous studies in which a decrease in this lymphocyte population in both blood and spleen was observed^23^. Treg cells are an essential subset in the maintenance of immune homeostasis and tolerance and their proportion was also inversely correlated with the performance in exhausted animals. Previous studies found that, in contrast to moderate exercise training^60^, intensive training could decrease the number of circulatory Treg in both animal models^61^ and marathon runners^62^. This decrease was attributed at least partially to the higher cortisol production observed after exhaustion as we previously observed in similarly trained rats^23^, since an in vitro study demonstrated that dexamethasone (synthetic glucocorticoid) exposure for 24 h decreases FoxP3+ expression in peripheral blood mononuclear cells^63^. On the other hand, unlike what has been previously described in spleen and blood^23^, MLN NK cell proportion was not modified by either intensive training or the final exhaustion.

Regarding the predominant lymphocyte populations, the intensive training programme and possible overtraining increased the proportion of Tαβ+ lymphocytes -and above all, Th cells since the Tc proportion was significantly lower—while it decreased that of B cells. Nevertheless, after the final exhaustion test, both B and T cell proportions were similar to that in sedentary rats. Other studies have reported a reduced T cell proportion in mouse submandibular lymph nodes after carrying out an intensive treadmill run test^24^. It could be hypothesized that with exhaustion, MLN Th cells move to the blood thus decreasing (normalizing) their proportion in the MLN; however, we have previously described a reduction in both blood Th and Tc cell proportions due to exhaustion^23^. In this case, these changes might be explained by a redistribution of lymphocytes within other lymphoid and non-lymphoid organs^25^ and the lymphocyte apoptosis^64,65^ induced by the release of catecholamines and glucocorticoids due to the intensive exercise stress. In this regard, a previous study in animals with a similar training program showed higher cortisol levels ^23,26^.

Apart from the changes in MLN T and B lymphocyte proportions, intensive training and exhausting exercise also modified their functionality. In the case of T lymphocytes, the intensive training programme, possibly inducing overtraining, decreased their proliferation capacity. Previous research has already reported the reduced lymphocyte proliferation capacity induced by intensive training^66^ and even acute bouts of exercise of a wide range of intensities^67^. This decrease could be partially due to the lymphopaenia and the higher cortisol levels found in rats which were similarly trained^23,26^, since glucocorticoids’ immunosuppressant properties target both cell trafficking and proliferation capacity^67,68^. On the other hand, the well-characterized exhausting exercise-induced acute lymphocytosis^49,50^, which we previously reported^26^, together with the increase in IL-2 secretion by MLN lymphocytes observed in the current study and by other authors^69,70^, may explain the sudden increase in proliferation right after performing the final exhaustion test.

With regard to other cytokines released by stimulated MLNLs, we observed an increase in IFN-γ production by lymphocytes from trained animals, which was even higher in rats who performed the final exhaustion test. The IFN-γ/IL-4 ratio (Th1/Th2 balance) was also higher in exhausted animals, showing a Th1 bias in MLNs, probably evidencing a local inflammatory process. Concerning IL-10 secretion, it positively correlated with the performance achieved in the final exhaustion test, again evidencing an intensity and/or running length-dependent exercise-induced immune disruption. The exhaustion-induced cortisol increase we previously reported^23^ could explain this association, since an in vitro study reported an increase in IL-10 expression in peripheral blood mononuclear cells after 24 h culture with dexamethasone^63^.

Finally, in order to assess the functionality of MLN B lymphocytes, IgA, IgM and IgG concentrations in cell supernatants were quantified. There was a higher MLN production of IgG due to both the intensive training and exhaustion, although these levels were lower 24 h later. There were no changes in IgA and IgM production due to exercise. These findings are in line with previously reported results in serum^23^. We hypothesized that, as previously reported in spleen^23^, MLN could be sensitive to the repetitive stress induced by the intensive training programme, which might upregulate the glucocorticoids and adrenergic receptors^71^ and this, in addition to the higher cortisol levels observed in similarly exercised animals^23^, may explain the higher in vitro production of IgG by MLN lymphocytes.

In summary, intensive training for 5 weeks in female Wistar rats, followed or not by an additional exhaustion test, appears to have caused an overtraining condition which modified both mucosal immunity and the intestinal epithelial barrier integrity. In particular, the intensive training programme decreased the salivary IgA concentration, impaired the claudins and occludin gene expression in the small intestine and altered the mesenteric lymph node lymphocyte composition while decreasing their proliferation capacity and increasing their IFN-γ secretion. The final exhaustion enhanced the caecal production of IgA while it impaired the zonula occludens expression and enhanced the IL-2 and IFN-γ secretion by mesenteric lymph node lymphocytes. These findings could partially explain the decline of the mucosal immunity and the gastrointestinal symptomatology induced by intensive exercise and in overtrained athletes. Further research might clarify whether a more intensive or longer exercise training can exacerbate the observed alterations and search for accurate nutritional strategies to counteract and even prevent them.

## Methods

### Animals

Female Wistar rats (3 week-old at arrival) were provided by Envigo (Huntingdon, United Kingdom) and were maintained at the animal facility of the Faculty of Biology (University of Barcelona). Female rats were used because previous studies reported a better adaptation to treadmill running than male rats^34,73^ while the impact of exercise on immunological variables was not influenced by gender^73^. The animals were kept under controlled conditions of temperature and humidity, in a 12 h/12 h light/dark cycle. Animals (2–3 per cage) were given ad libitum access to food (Teklad Global 14% Protein Rodent Maintenance Diet, Teklad, Madison, WI, USA) and water. Body weight (BW) and food intake were monitored throughout the study. Animal procedures were approved both by the Ethical Committee for Animal Experimentation of the University of Barcelona (CEEA/UB ref. 464/16) and the Catalonia Government (DAAM 9257). The number of animals was established according to the minimum required for providing statistically significant differences among groups, using the Appraising Project Office’s programme from the Universidad Miguel Hernández de Elche (Alicante, Spain). Moreover, the number of rats in each group was adjusted following the University Ethical Committee guidelines and applying the three Rs rule for experimenting in animals. All methods were carried out in accordance with relevant guidelines and regulations.

### Training programme

After a 2-week adaptation period, rats were submitted to a high-intensity exercise training (n = 24) or remained as a sedentary control group (n = 8; SED). A high-intensity training was induced in rats by running in a treadmill (LE8700, Panlab, Harvard, USA, and Exer3/6 treadmill Columbus, Ohio, USA) 5 days per week for 5 weeks, involving two exhaustion tests per week, as previously reported^23,26^. Briefly, every Monday and Friday, the exercised group carried out an exhaustion test, which consisted of running 15 min at 60% of the maximum speed average achieved in the previous Monday’s exhaustion test (the speed of the first Monday’s exhaustion test was 30 m × min^−1^), and from then on, the speed was progressively increased until exhaustion. On Tuesday, Wednesday and Thursday, rats trained for 20, 25 and 30 min, respectively, at 60% of the maximum speed average achieved in the previous Monday’s exhaustion test. At the end of the training period, runner animals were distributed into 3 groups (n = 8) with a similar ability to run: trained group (T, whose samples were obtained 24 h after a regular training), exhausted group (TE, whose samples were obtained immediately after an additional final exhaustion test) and 24 h post-exhaustion group (TE24, whose samples were obtained 24 h after the additional final exhaustion test). The SED group was exposed to the same conditions of maintenance and isolation stress as runner rats. As a positive reinforcement, both runner and SED rats received at the end of training or isolation, a 50% solution of condensed milk (100 μL/100 g BW).

### Sample collection and processing

At the end of the study, animals were anaesthetized by intramuscular injection of ketamine (Merial Laboratories S.A. Barcelona, Spain) and xylazine (Bayer A.G., Leverkusen, Germany) (90 mg × kg^−1^ and 10 mg × kg^−1^, respectively) and exsanguinated. The MLN, small intestine, SMG and CC were collected.

The lymphocytes from MLN were isolated in aseptic conditions by passing the tissue through a 40 µm sterile mesh cell strainer (Thermo Fisher Scientific, Barcelona, Spain), as previously detailed^72^. MLNL counting and viability were determined by a Countess Automated Cell Counter (Invitrogen, Thermo Fisher Scientific).

A 0.5 cm portion of the middle part of the small intestine was immediately kept in RNAlater (Ambion, Life Technologies, Austin, TX, USA) and stored at − 20 °C until the determination of gene expression of some molecules by Real-Time Polymerase Chain Reaction (RT-PCR). The distal part of the small intestine was used to obtain GW, as established previously in our laboratory^73^. Briefly, it was flushed with cold PBS (pH 7.2) to remove faecal content, opened lengthwise, cut into 1–2 cm pieces, weighed and incubated with 3 mL of PBS for 10 min in a shaker at 37 °C (55 shakings × min^−1^). After centrifugation (538 g, 4 °C, 10 min), supernatants were collected and stored at − 20 °C until a1AT and IgA quantification.

SMG and CC homogenates were obtained using a tissue homogenizer (Polytron, Kinematica, Lucerne, Switzerland and Pellet Pestle Cordless Motor, Kimble, Meiningen, Germany, respectively), as described in previous studies, and kept at − 20 °C until IgA quantification^74^.

### MLNL phenotypic analysis

MLNLs (5 × 10^5^ cells) were extracellularly and intracellularly stained by using mouse anti-rat monoclonal antibodies (mAb) conjugated to fluorescein isothiocyanate (FITC), phycoerythrin (PE), peridinin-chlorophyll-a protein (PercP), allophycocyanin (APC) and brilliant-violet 421 (BV421), as previously described^72^. The following fluorochrome-conjugated mAb antibodies were used: FITC-TCRαβ, FITC-CD8β, FITC-CD25, PE-CD161a, PE-TCRγδ, PE-CD4, PerCP-CD8α, APC-CD4, and BV421-CD45RA (BD Biosciences, Madrid, Spain) and APC-FoxP3 (eBioscience, Frankfurt, Germany). For extracellular staining, MLNL were incubated with saturating amounts of mAb in PBS containing 2% FBS and 0.1% NaN_3_ (darkness, 4 °C, 20 min). For intracellular staining, cells previously labelled extracellularly with anti-CD4-PE and anti-CD25-FITC mAb were treated with Foxp3 fixation/permeabilization kit (eBioscience). Then, intracellular staining with anti-Foxp3-APC mAb was carried out (darkness, 4 °C, 30 min), as described in previous studies^75^. All stained cells were fixed with 0.5% p-formaldehyde and stored at 4 °C in darkness until analysis by flow cytometry. A negative control staining without any mAb antibody and a staining control for each mAb were included. Analyses were performed using a Gallios Cytometer (Beckman Coulter, Miami, FL, USA) in the Flow Cytometry Unit of the Scientific and Technological Centres of the University of Barcelona (CCiT-UB) and by Flowjo v10 software (Tree Star, Inc., Ashland, OR, USA). Changes in lymphocyte phenotype by exercise are represented considering the SED group mean value as 1, therefore, all values are expressed as a fold change of the mean value with respect to the SED group.

### MLNL stimulation and proliferation

MLNL (10^5^ cells/well) were incubated in quadruplicate in 96-well plates (TPP, Sigma-Aldrich, Madrid, Spain) and stimulated or not with ConA (5 µg × mL^−1^, Sigma-Aldrich) for 48 h. T cell proliferation was quantified using a BrdU Cell Proliferation Assay kit (MerckMillipore, Darmstadt, Germany), according to manufacturer’s instructions. The proliferation rate was calculated by dividing the optical density of ConA stimulated cells with the optical density of non-stimulated cells. Changes in MLNL proliferative capacity by exercise are represented considering the SED group mean value as 1.

Moreover, after 48 h of incubation, supernatants from ConA-stimulated and non-stimulated conditions were collected and stored at − 80 °C in order to evaluate the cytokine production. Supernatants from non-stimulated conditions were also used to quantify IgA, IgM and IgG.

### Cytokine quantification

The production of IFN-γ, IL-2, IL-4 and IL-10 was determined in MLNL supernatants by ProcartaPlex Multiplex Immunoassay (Affymetrix, eBioscience, San Diego, USA), according to the manufacturer’s protocol. Data were acquired by MAGPIX Cytometer (Affymetrix) in the CCiT-UB and analysed by ProcartaPlex Analyst v1.0 software (Affymetrix). The lower limits of detection were as follows: 3.34 pg × mL^−1^ for IFN-γ; 1.82 pg × mL^−^1 for IL-2; 0.62 pg × mL^−^1 for IL-4; 6.01 pg × mL^−^1 for IL-10. The ratio of mean fluorescence intensity (MFI) obtained from cells supernatants under ConA stimulation with respect to the non-stimulated condition was calculated. Changes in MLNL cytokine profile by exercise are expressed considering the SED group mean value as 1.

### Immunoglobulin quantification

The concentrations of IgG, IgM and IgA in MLNL culture supernatants, GW, SMG or CC homogenates were quantified by a sandwich ELISA (Bethyl Laboratories Inc., Montgomery, TX, USA). After assay development as previously described^76^, absorbance was measured on a microplate photometer (Labsystems Multiskan, Helsinki, Finland) and data were analysed by Ascent v.2.6 software (Thermo Fisher Scientific, S.L.U, Barcelona, Spain) according to the respective standard curves. The immunoglobulin content in SMG and CC was normalized by total protein concentration which was measured using the Pierce-660 nm ready-to-use Protein Assay Reagent (Thermo Fisher Scientific). Changes in each Ig concentration are expressed considering the SED group mean value as 1.

### Gene expression in small intestine by real-time polymerase chain reaction (RT-PCR)

The intestinal expression of occludin, claudin-2, claudin-4, ZO-1, ZO-2, pIgR, TGF-β1, RAR-α and CCL25 was assessed in the small intestine. This tissue, kept in RNA later, was homogenized in a lysing matrix tube (MP Biomedicals, Illkirch, France) by a FastPrep-24 instrument (MP Biomedicals) for 30 s. Intestinal RNA was then isolated with the RNeasy mini kit (Qiagen, Madrid, Spain) following the manufacturer’s instructions. RNA quantification and purity were assessed using a NanoPhotometer (BioNova Scientific S.L., Fremont, CA, USA). RNA was reverse-transcribed in a thermal cycler (PTC-100 Programmable Thermal Controller, BioRad, Hercules, CA, USA) using TaqMan Reverse Transcription Reagents (Applied Biosystems, AB, Weiterstadt, Germany) in order to obtain the corresponding cDNA.

The RT-PCR was carried out in duplicate for each sample using the ABI Prism 7900 HT quantitative RT-PCR system (AB) with the following specific PCR TaqMan primers and probes (AB): Ocln (Rn00580064_m1, Inventoried, I); Cldn2 (Rn02063575_s1, I); Cldn4 (Rn01196224_s1, I); ZO1 (Rn02116071_s1, I); ZO2 (Rn01501483_m1, I); Pigr (Rn00562362_m1, I); Tgfb1 (Rn00572010_m1, I); Rara (Rn00580551_m1,I); and Ccl25 (Rn01403352_m1, I). SDS version 2.4 software (AB) was used to assess the gene expression. The gene expression of the target genes was normalized with respect to the housekeeping genes *Gusb* (β-glucuronidase, Rn00566655_m1, I) or *β-actin* (Rn00667869_m1, I), depending on their expression level, using the 2^-ΔΔCt^ method^77^. For *Cldn2* and *ZO-1* the gene expression of *Gusb* was used as housekeeping gene, whereas for the rest of the genes, *β-actin* expression was used. Changes in small intestine gene expression are expressed considering the SED group mean value as 1.

### Small intestine permeability

The concentration of a1AT in GW was assessed as a marker of intestinal protein loss and mucosal permeability. The quantification was performed with the ELISA Kit for rat a1AT (Cloud-CloneCorp., Houston, TX, USA) following the manufacturer’s instructions. Absorbance was measured on a microplate photometer (LabSystems Multiskan) and data were interpolated by Ascent v.2.6 software (Thermo Fisher Scientific) according to the standard, which ranged from 500 to 7.8 ng × mL^−1^. Changes in GW a1AT concentration are expressed considering the SED group mean value as 1.

### Statistical analysis

Statistical analysis of the data was carried out using IBM Social Sciences Software Program (SPSS, version 26.0, Chicago, IL, USA). Variance equality and normality of the data was tested by Levene’s and Shapiro–Wilk’s test, respectively. A one-way ANOVA test was applied and, if significant differences were detected, Tukey’s post hoc test was performed. Kruskal–Wallis test was used when results were neither equally nor normally distributed, followed by Mann–Whitney U test in the case of significant differences among groups. The Spearman correlation coefficient was used to assess correlations between the variables analysed and the performance achieved. When comparing variables from two groups throughout the study, the repeated measures ANOVA test was used to assess whether there was a significant interaction between time (day of study) and exercise condition (SED or runner). Once we confirmed there was a significant interaction (p < 0.05 in Greenhouse–Geisser test, Huynh–Feldt test and Lower-bound test), we performed the unpaired Student’s t test to detect between which days of study the differences between SED and runner rats were statistically significant. To compare variables during the study (e.g., maximum time lasted in the exhaustion tests), a repeated-measures ANOVA was applied followed by paired Student’s t test. Significant differences were considered when p < 0.05.
